# Targeted Development and Validation of Clinical Prediction Models in Secondary Care Settings: Opportunities and Challenges for Electronic Health Record Data

**DOI:** 10.2196/57035

**Published:** 2024-10-24

**Authors:** I S van Maurik, H J Doodeman, B W Veeger-Nuijens, R P M Möhringer, D R Sudiono, W Jongbloed, E van Soelen

**Affiliations:** 1 Northwest Academy, Northwest Clinics Alkmaar, Pr Julianalaan 14, Alkmaar, 1815JE, Netherlands, 31 0880853821; 2 Department of Information and Communication Technology, Northwest Clinics Alkmaar, Alkmaar, Netherlands; 3 Department of Radiology, Northwest Clinics Alkmaar, Alkmaar, Netherlands; 4 Department of Clinical Chemistry, Hematology and Immunology, Northwest Clinics Alkmaar, Alkmaar, Netherlands

**Keywords:** clinical prediction model, electronic health record, targeted validation, EHR, EMR, prediction models, validation, CPM, secondary care, machine learning, artificial intelligence, AI

## Abstract

Before deploying a clinical prediction model (CPM) in clinical practice, its performance needs to be demonstrated in the population of intended use. This is also called “targeted validation.” Many CPMs developed in tertiary settings may be most useful in secondary care, where the patient case mix is broad and practitioners need to triage patients efficiently. However, since structured or rich datasets of sufficient quality from secondary to assess the performance of a CPM are scarce, a validation gap exists that hampers the implementation of CPMs in secondary care settings. In this viewpoint, we highlight the importance of targeted validation and the use of CPMs in secondary care settings and discuss the potential and challenges of using electronic health record (EHR) data to overcome the existing validation gap. The introduction of software applications for text mining of EHRs allows the generation of structured “big” datasets, but the imperfection of EHRs as a research database requires careful validation of data quality. When using EHR data for the development and validation of CPMs, in addition to widely accepted checklists, we propose considering three additional practical steps: (1) involve a local EHR expert (clinician or nurse) in the data extraction process, (2) perform validity checks on the generated datasets, and (3) provide metadata on how variables were constructed from EHRs. These steps help to generate EHR datasets that are statistically powerful, of sufficient quality and replicable, and enable targeted development and validation of CPMs in secondary care settings. This approach can fill a major gap in prediction modeling research and appropriately advance CPMs into clinical practice.

## Background

In health care, distinct tiers of care, namely primary, secondary, and tertiary care, play vital roles in addressing patients’ diverse medical needs. Patients requiring specialized medical attention or hospital care are generally treated in secondary care settings. Approximately one-third of primary care patients are referred to secondary care, and the majority of these patients are treated and monitored in this setting [1]. Tertiary care consists of highly specialized services for highly complex diseases. Less than 5% of patients require care in a tertiary setting. The distribution of patients across primary and secondary care settings may differ between countries and health care systems; some countries require a referral from primary care to enter secondary care, while in other countries patients have direct access to medical specialists without a referral. While there can be significant variability in primary and secondary care structures, variablity in tertiary care structures are generally less pronounced. This is because tertiary care focuses on highly specialized and complex conditions that are often standardized based on international research and protocols. Academic hospitals and specialized centers provide similar highly specialized care worldwide.

Due to the complexity of care, strong research facilities, and involvement in clinical trials, most clinical understanding and knowledge of medical conditions come from patients treated in tertiary settings [2,3]. Similarly, in this setting many clinical prediction models (CPMs) are developed. A CPM is a statistical or artificial intelligence-based tool used in health care to predict future health events in individual patients using a set of predictors or risk factors. CPMs have the potential to combine and weigh large amounts of patient information, enabling the stratification of patients based on their risk of future health events. This informs decision-making processes and may guide the allocation of resources and interventions. While such models are also developed in primary and secondary settings, CPMs developed in tertiary settings may have great potential to be useful in secondary care, where the patient case mix is broad and practitioners need to triage patients efficiently.

However, the usefulness of such CPMs depends significantly on their quality in the population of intended use. Recent discussions emphasize the importance of targeted validation, which is the assessment of a CPM’s quality in the specific population for which it is intended. Yet, this specification of the population of intended use is often lacking in publications [4]. Secondary health care settings, where large numbers of patients with specialized medical needs are treated, accumulate vast amounts of data in electronic health records (EHR) on a daily basis. Despite this potential, CPMs are often not developed or validated on data from secondary care populations due to the scarcity of appropriate datasets. This is known as the “validation gap.” In this viewpoint, we discuss the opportunities and challenges faced when considering EHR data from secondary health care settings for the development or validation of CPMs.

## Importance of Targeted Validation of CPMs

The performance of CPMs is significantly influenced by the case mix of patients (ie, baseline characteristics of the patients) and the prevalence of the outcome [5-7]. The case mix of a secondary care population is essentially different from a tertiary care population. Due to these case mix differences, a CPM developed in tertiary care often performs poorly in secondary care populations [8].

For instance, in cardiovascular risk prediction models, such disparities in patient characteristics and outcomes between tertiary and secondary care settings substantially impact model performance. Research by Wynants et al [3] highlights the challenges of model transportability and generalizability. In a review, they showed that 23 out of 50 studies did not describe the population of intended use. In those studies that reported health care setting, all participating centers were in tertiary or academic settings. One of the studies applied a tertiary CPM in a secondary care setting. In this secondary care setting, patients were older, the outcome was less prevalent, and patients more often had (multiple) risk factors such as diabetes and hypertension. Under these circumstances, the CPM severely overestimated event probabilities when applied to secondary care. Similarly, in chronic obstructive pulmonary disease management, the use of CPMs is complicated by variations in patient profiles across health care settings. While primary and secondary care cohorts exhibit marked heterogeneity in health status, tertiary care cohorts tend to comprise more homogeneous samples [2].

These examples, along with many others in medical literature [9,10], demonstrate poor model performance, specifically poor calibration, of tertiary CPMs in the population of intended use. Arguably, these prediction models are most useful at lower levels of care, where the patient case mix is broad and practitioners need to triage patients efficiently [11]. More concretely, an overestimation of event probabilities means that patients could be incorrectly categorized as high-risk based on a CPM that is poorly calibrated to the target population. Such inaccurate risk prediction can be misleading and may negatively influence clinical practice; it may lead to false expectations from the patient or professional, or patients may make personal decisions in anticipation (or absence) of an event [12]. CPM specialists argue that poor calibration may render an algorithm less clinically useful than a competing model with lower discriminative ability but is well calibrated [9].

While checklists exist to improve reporting quality [13,14] and assess the risk of bias [15] in CPM development and validation, targeted validation remains an uncommon practice. Sperrin et al [4] rightly argue that we should report the intended population of use more explicitly. This means that if, for example, a CPM is intended to aid decision-making in a secondary care setting in the Netherlands, then it should be developed and validated in a secondary care setting in the Netherlands. Such targeted validation requires data from the population of intended use. The difficulty lies in the scarcity of structured or rich datasets from secondary settings available to assess the quality of a CPM. Addressing this validation gap remains a challenge in CPM literature and hampers the implementation of CPMs in clinical practice, emphasizing the importance of leveraging EHR data from secondary health care settings for CPM development and validation.

### EHR Datasets and Text Mining Tools

Every day, hospitals collect an enormous amount of health information in EHRs. Data in these EHRs have structured and unstructured formats. Structured EHR data comprise data in fixed numerical or categorical areas, such as diagnoses, prescriptions, and laboratory values, while unstructured data includes clinical documentation such as notes, referral letters, or discharge summaries produced by health care personnel [16]. These documents are inputted as free text into EHRs and offer a complete picture of a patient’s condition. It is estimated that more than 70% of EHR data is stored as free text. Even information that seems structured, such as a total score from a questionnaire, is often stored as free text in the EHR in letters or notes. To conduct good research in general, this data should be converted into structured formats and datasets. Specifically, to validate a CPM, it is required that a certain predictor, which is part of the CPM, is collected and recorded in a consistent manner. Leveraging the value of unstructured data is key to generating meaningful insights from clinical data [16-18].

Text mining tools and natural language processing (NLP) techniques allow us to transform unstructured documents into a structured format to enable analysis and the generation of high-quality CPMs [19]. Text mining applications are increasingly used in research and computational settings, but are now also commercialized in software applications (for example, CTcue from IQVIA or Amazon Comprehend Medical from Amazon Web Services) that allow hospitals to generate structured data and subsequently cohorts of patients with a specific disease more efficiently from their electronic medical files.

With respect to targeted development and validation of CPMs, specific predictors can be found more easily, especially when the CPM is based on commonly used clinical measures and data. These datasets are often very large and are therefore statistically powerful [16].

## EHR Data Quality

### Overview

Leveraging EHR data for research brings challenges with regard to data quality. These records are prone to ascertainment bias and missingness [18,20], especially concerning free text data, where semantic and context understanding are required to correctly classify types of information. Furthermore, data quality depends on how and if a clinician records information in the EHR [19]. This may be even more problematic in secondary teaching hospitals, which have a higher turnover of personnel. Another challenge is information overload, which poses a substantial problem in accessing a particular, significant piece of information from vast datasets. A recent systematic review shows additional technical challenges such as lack of labeled data, spelling correction, medical abbreviation, negation detection, and clinical entity recognition [21].

Data quality is a key contributor to the quality and success of developed CPMs: “rubbish in, rubbish out.” While NLP software is developing rapidly and their quality improves, their output needs to be checked and validated carefully. When using EHR data for the development and validation of CPMs, alongside the widely accepted checklists, we propose additionally considering the following steps in the data extraction process:

### Step 1: Include a Clinician, Nurse, or Health Care Professional as the Local EHR Expert and in the EHR Data Extraction Process

This is not always the case, as data extraction may be conducted by supporting staff, business intelligence specialists, or students and interns. However, clinicians have firsthand knowledge of their patients’ conditions, treatments, and histories. Clinicians and health care professionals may be aware of certain patient details that are not well-documented in the EHR, such as informal diagnoses or symptoms not coded in the system. Including this information helps create a more comprehensive and accurate dataset, informing the EHR data capturing process. With regard to unstructured data; discuss the clinical workflow and how and when specific clinical notes are made. As a simple example: when extracting data from the “medical history” part of medical notes, you might find “Hypertension: -.” Does this mean that information on hypertension for this patient is missing, is not applicable, or is absent?

With regard to structured data, check (if applicable) whether protocol changes occurred in the period of interest. Unlike research databases, major protocol changes are not documented in EHRs. In a hospital setting, system updates are regularly performed, new equipment is purchased, or measurement methods are changed. This is not documented in the EHR of individual patients. When using EHR data for research purposes, such as developing or validating a CPM, these organizational factors should be considered. For example, if the clinical chemistry laboratory first measured thyroid hormone FT4 with a Beckman Coulter analyzer with normal values between 7‐16 pmol/L and later switched to Siemens with normal values between 11‐21 pmol/L, this significantly influences the outcome of CPMs including FT4. Another example is the measurement of the tumor marker carcinoembryonic antigen, where levels of carcinoembryonic antigen measured with Siemens are approximately 25% lower compared to those measured with Beckman Coulter. Harmonization of such laboratory results within a hospital, but also between hospitals, is therefore important and requires knowledge of protocol changes over time.

### Step 2: Perform Validity Checks on the Generated Dataset

Data validation and verification are broadly accepted exercises in research settings. It is the process of checking whether entered data is accurate and consistent. This may encompass the crosschecking of data in a random set of cases, which may be even more relevant in research where data is derived from EHRs. EHR data are complex and heterogeneous, originating from different systems, formats, and medical practices. This variability can introduce inconsistencies and errors. Validation and verification processes are essential to standardize the data, correct inconsistencies, and ensure uniformity in the data used for research.

In addition to checking the data quality of specific variables extracted from EHRs, we advise also executing a crosscheck on the included cases. Specifically, if software is used to compose a cohort, let a clinician provide a list of patients that they believe should be included in the generated dataset, and check whether that is indeed the case (ie, do I find the cases that I should find?). This is important for a number of reasons. First, clinicians can identify patients who meet specific criteria based on nuances that may not be captured in the EHR data alone. This clinical insight is invaluable for ensuring that the correct patients are included in the research cohort. Second, automated systems rely on predefined algorithms to identify patients, but these algorithms can sometimes miss relevant cases or include irrelevant ones. Lastly, clinicians can provide supplementary information to fill gaps in the EHR data, enhancing the completeness and richness of the dataset. This additional information can improve the robustness of the research outcomes.

### Step 3: Deliver Information or Metadata on How Certain Variables Are Constructed

Information should be provided and made publicly available on whether a variable is composed from structured codes or from a search in unstructured free-text (for example, reports) and include a list of search terms used (or excluded). Delivering detailed information or metadata on how certain variables are constructed, and understanding whether these variables come from structured or unstructured electronic patient record data, enhances data quality and integrity during the data extraction process and is crucial for transparency and reproducibility [22]. Knowing whether a variable comes from structured (eg, coded fields or predefined formats) or unstructured (eg, free-text notes or narratives) data is essential as they have different characteristics. Structured data is generally more reliable, easier to analyze, and in some cases similar across hospitals (eg, Anatomical Therapeutic Chemical Classification System codes). It follows a predefined format, making it straightforward to extract and use in statistical analyses. Unstructured data, on the other hand, is rich in detailed information but more challenging to analyze due to variability and complexity. NLP and other sophisticated methods are often required to extract meaningful information from unstructured data. Clear documentation of variable construction enhances the impact and credibility of research findings, making it easier for clinicians and policy makers to apply the results in real-world settings.

## Conclusion

CPMs may be particularly valuable in secondary care settings, and the introduction of software applications for text mining of EHRs allows the generation of structured “big” datasets. However, the imperfection of EHRs as a research database requires careful validation of data quality. On using EHR data for the development and validation of CPMs, alongside the widely accepted checklists, we propose to additionally consider three practical steps: (1) let a local EHR expert (clinician or nurse) be involved in the data extraction process, (2) perform validity checks on the generated datasets, and (3) provide metadata on how variables were constructed from EHRs. If successful, such datasets are statistically powerful and enable targeted development and validation of CPMs in secondary care settings, filling a major gap in prediction modeling research.

## Abbreviations

CPM: clinical prediction model
EHR: electronic health record
NLP: natural language processing
